# San-Huang-Xie-Xin-Tang Protects against Activated Microglia- and 6-OHDA-Induced Toxicity in Neuronal SH-SY5Y Cells

**DOI:** 10.1093/ecam/nep025

**Published:** 2011-01-04

**Authors:** Yu-Tzu Shih, Ing-Jun Chen, Yang-Chang Wu, Yi-Ching Lo

**Affiliations:** ^1^Graduate Institute of Natural Products, Kaohsiung Medical University, Kaohsiung 807, Taiwan; ^2^Department of Pharmacology, College of Medicine, Kaohsiung Medical University, Kaohsiung 807, Taiwan

## Abstract

San-Huang-Xie-Xin-Tang (SHXT), composed of *Coptidis rhizoma*, *Scutellariae radix* and *Rhei rhizoma*, is a traditional Chinese herbal medicine used to treat gastritis, gastric bleeding and peptic ulcers. This study investigated the neuroprotective effects of SHXT on microglia-mediated neurotoxicity using co-cultured lipopolysaccharide (LPS)-activated microglia-like BV-2 cells with neuroblastoma SH-SY5Y cells. Effects of SHXT on 6-hydroxydopamine (6-OHDA)-induced neurotoxicity were also examined in SH-SY5Y cells. Results indicated SHXT inhibited LPS-induced inflammation of BV-2 cells by downregulation of iNOS, NO, COX-2, PGE_2_, gp91^phox^, iROS, TNF-*α*, IL-1*β*, inhibition of I*κ*B*α* degradation and upregulation of HO-1. In addition, SHXT increased cell viability and down regulated nNOS, COX-2 and gp91^phox^ of SH-SY5Y cells co-cultured with LPS activated BV-2 cells. SHXT treatment increased cell viability and mitochondria membrane potential (MMP), decreased expression of nNOS, COX-2, gp91^phox^ and iROS, and inhibited I*κ*B*α* degradation in 6-OHDA-treated SH-SY5Y cells. SHXT also attenuated LPS activated BV-2 cells- and 6-OHDA-induced cell death in differentiated SH-SY5Y cells with db-cAMP. Furthermore, SHXT-inhibited nuclear translocation of p65 subunit of NF-*κ*B in LPS treated BV-2 cells and 6-OHDA treated SH-SY5Y cells. In conclusion, SHXT showed protection from activated microglia- and 6-OHDA-induced neurotoxicity by attenuating inflammation and oxidative stress.

## 1. Introduction

Inflammation appears to be a complicating factor in many age-related neurodegenerative diseases, such as Parkinson's disease (PD) [1], Alzheimer's disease (AD) [2] and multiple sclerosis [3]. Microglia are thought to mediate the innate defense system in the central nervous system [4]. Activated microglia enhances pro-inflammatory and neurotoxic responses and neuronal injury [5, 6]. Microglia-mediated neurotoxicity was induced through release of pro-inflammatory cytokines such as tumor-necrosis factor alpha (TNF)-*α*, interleukin 1*β* (IL-1*β*), nitric oxide (NO) and prostaglandin E_2_ (PGE_2_), this yields enhanced reactive oxygen species (ROS) [7] and reactive nitric oxide species (RNS) [8, 9]. The deleterious consequences of excessive oxidation and pathophysiological role of ROS have been intensively studied in Alzheimer's disease. Studies indicated that anti-inflammatory or anti-oxidative stress drugs exert neuroprotective effects [10–13]. Therefore, anti-inflammatory agents and antioxidants are considered a promising approach to slowing the progression and limiting the extent of neuronal cell loss in these disorders.

Many herbal medicines and dietary supplements are sold as aids to improve memory, treat neurodegenerative diseases or create favorable effects on the central nervous system. Increasing evidence has suggested that traditional Chinese medicines or herbal extracts possess neuroprotective benefits through their distinct and multiple mechanisms, including anti-inflammation and anti-oxidation [14–19]. San-Huang-Xie-Xin-Tang (SHXT), a traditional Chinese medicinal formula containing *Coptidis rhizome* (*Coptis chinesis* Franch), *Scutellariae radix* (*Scutellaria baicalensis* Georgi) and *Rhei rhizome* (*Rheum officinale* Baill), has been used to treat gastritis, gastric bleeding and peptic ulcers [20]. The preventive effects of SHXT on lipopolysaccharide (LPS) and *Helicobacter pylori*-induced inflammatory responses have been confirmed by our previous studies [21–23]. Studies indicate that several components in SHXT show inhibitory effects on neurotoxicity. Baicalein and baicalin, the major flavonoids in *Scutellaria baicalensis*, can reduce beta amyloid protein-induced neurotoxicity [24]. Baicalein [14, 18] and wogonin [19] attenuate inflammation-mediated neurodegeneration [14, 18, 19]. Baicalin attenuates oxygen-glucose deprivation-induced injury in neurons [25]. Emodin, an anthraquinone derivative extracted from *Rhei rhizome*, decreases glutamate-induced neurotoxicity [26].

LPS is commonly used to investigate the impact of inflammation on neuronal death, and studies indicate that microglia are necessary for LPS-induced neurotoxicity [27, 28]. LPS is not toxic to neurons, but LPS stimulates glial cells to produce factors that are toxic to neurons [28–30]. On the other hand, the auto-oxidation of the neurotransmitter dopamine to 6-hydroxydopamine (6-OHDA) generates ROS and reactive quinones subsequently inducing cell death [31, 32]. Therefore, we investigated the effects of SHXT on LPS activated microglia-induced neurotoxicity by using the *invitro* model of co-culture microglia-like BV-2 cells with the neuronal-like neuroblastoma SH-SY5Y cell. We also examined the effects of SHXT on 6-OHDA-induced neurotoxicity in dopaminergic neuroblastoma SH-SY5Y cells.

## 2. Materials and Methods

### 2.1. Reagents

The voucher specimen and method for extraction and analysis of SHXT were described previously [22]. Briefly, a blended mixture of *Coptidis rhizome*, root of *Scutellariae radix* and rhizome of *Rhei officinale* Baill was prepared in a ratio of 1 : 1 : 2, respectively. The content of each component in SHXT is shown as follows (*μ*g/mL): baicalin 1153.07 ± 56.36, baicalein 82.81 ± 3.74, emodin 11.15 ± 1.22, wogonin 19.55 ± 0.83, rhein 126.12 ± 3.84, berberine 62.14 ± 4.27, coptisine 6.15 ± 0.34, palmatine 25.11 ± 3.78, sennoside A 128.02 ± 13.56 and sennoside B 95.90 ± 3.59. Dimethyl sulfoxide (DMSO), lipopolysaccharide (LPS, L8274) from *Escherichia coli* (O26:B6), 3-[4,5-dimethylthiazol-2-yl]-2,5-diphenyltetrazolium bromide (MTT), 2′,7′-dichloro-dihydrofluorescein diacetate (H_2_DCF-DA), 6-hydroxydopamine (6-OHDA), dibutyryl cyclic AMP (db-cAMP), mouse antibody against iNOS and *β*-actin were purchased from Sigma Aldrich (USA). Dulbecco's modified Eagle's medium (DMEM), fetal bovine serum (FBS), penicillin, amphotericin B and streptomycin were purchased from GIBCO/BRL Life Technologies (USA). Mouse antibody against gp91^phox^ and all materials for SDS-polyacrylamide gel electrophoresis were purchased from Bio-Rad (USA). Goat antibody against COX-2 and HO-1, mouse antibody against I*κ*B*α*, rabbit antibody against nNOS and all horseradish peroxidase-conjugated secondary antibodies were purchased from Santa Cruz Biotechnology (USA). Enhanced chemiluminescence agent was purchased from PerkinElmer Life and Analytical Sciences (USA). TNF-*α*, IL-1*β* and PGE_2_ enzyme immunoassay kits and NE-PER Nuclear and Cytoplasmic Extraction Reagents were purchased from Pierce Endogen (USA). TransFactor Family Colorimetric Kit NF-*κ*B was purchased from BD Bioscience, Clontech (USA). All other chemicals were purchased from Sigma Chemical Company (USA).

### 2.2. Microglial BV-2 Cell Culture and Activated Microglia with LPS

Mouse microglial cell line BV-2 was cultured in DMEM containing 10% (v/v) heat-inactivated FBS, 4 mM glutamine, 100 U/mL penicillin, 100 mg/mL streptomycin and 0.25 mg/mL amphotericin B at 37°C in a humidified incubator under 5% CO_2_ and 95% air. For the purpose of experiment, BV-2 cells were plated in 6-, 24- or 96-well sterile plates (1 × 10^5^ cells/mL). Then, cells were stimulated with LPS alone (100 ng/mL) or LPS with different concentrations of SHXT.

### 2.3. Human SH-SY5Y Neuroblastoma Cell Culture

The human neuroblastoma cell line SH-SY5Y (ATCC CRL-2266) was cultured in a medium consisting of a 1 : 1 mixture of DMEM and Ham's F-12 medium containing 10% heat-inactivated FBS, 4 mM glutamine, 100 U/mL penicillin, 100 mg/mL streptomycin and 0.25 mg/mL amphotericin B at 37°C in a humidified incubator under 5% CO_2_ and 95% air. In order to investigate the neuroprotective effect of SHXT on differentiated neuronal cell, SH-SY5Y cells were treated with 1 mM db-cAMP, a permeable analog of cAMP, to induce neuronal differentiation. SH-SY5Y cells (1 × 10^5^ cells/mL) were plated in 96-well sterile plates and db-cAMP was dissolved in distilled water and added to each well at a concentration of 1 mM in DMEM containing 10% FBS. The cells were then incubated for 4 days at 37°C, with the medium refreshed daily. Then, cells were treated with 6-OHDA alone (100 *μ*M) or 6-OHDA with different concentrations of SHXT for 24 h.

### 2.4. Co-Culture of SH-SY5Y Cells with Microglial BV-2 Cells

The effects of SHXT on microglia-mediated and inflammation-associated neuronal damage were determined using LPS stimulated BV-2 cells as a model of activated microglia [11]. Non-differentiated or differentiated SH-SY5Y cells were plated in 6- or 24-well plates (1 × 10^5^ cells/mL) and BV-2 cells were seeded onto cell culture inserts (pore size of 0.2 *μ*m; NUNCA/S, Roskilde, Denmark). Then the inserts were placed in the wells where SH-SY5Y cells were growing in DMEM containing 10% (v/v) heat-inactivated FBS, 4 mM glutamine, 100 U/mL penicillin, 100 mg/mL streptomycin and 0.25 mg/mL amphotericin B at 37°C in a humidified incubator under 5% CO_2_ and 95% air. The BV-2 and SH-SY5Y co-cultured cells were separated by filters present in the insert. In co-culture conditions, BV-2 cells were then stimulated with LPS (100 ng/mL) or LPS + SHXT for 24 h.

### 2.5. Cell Viability Assay

Cell viability was measured by a quantitative colorimetric assay with MTT, showing mitochondrial activity of living cells. In mono-culture, BV-2 or SH-SY5Y cells in 96-well plates were incubated with 25–200 *μ*g/mL of SHXT for 24 h. In 6-OHDA-induced neurotoxicity, non-differentiated or differentiated SH-SY5Y cells were treated with or without 6-OHDA (100 *μ*M) or 6-OHDA + SHXT for 24 h. Under co-culture conditions, BV-2 cells were treated with or without LPS (100 ng/mL) or LPS + SHXT for 24 h. After incubation, inserts containing BV-2 cells were removed. Non-differentiated or differentiated SH-SY5Y cells were incubated with MTT at a final concentration of 0.5 mg/mL for 3 h at 37°C. The reaction was terminated by addition of 200 *μ*L of DMSO. The amount of MTT formazon product was then determined by measuring the absorbance at 560 nm using a microplate reader.

### 2.6. Nitrite Measurement

Production of nitric oxide (NO) was determined by measuring the level of accumulated nitrite, a metabolite of NO in the culture supernatant using Griess reagent (1% sulfanilamide and 0.1% *N*-(1-naphthyl)ethylenediamide in 5% phosphoric acid). After 24 h of treatment with LPS (100 ng/mL) alone or LPS + SHXT, the culture supernatants were collected and mixed with an equal volume of Griess reagent and incubated at room temperature for 10 min. The absorbance was measured at 540 nm (OD_540_).

### 2.7. Enzyme-Linked Immunosorbent Assay (ELISA)

The productions of IL-1*β*, PGE_2_ and TNF-*α* in supernatant were detected using ELISA kits from Endogen (USA) according to the manufacturer's instructions. IL-1*β* and PGE_2_ in the supernatant were determined after 24 h of LPS treatment. TNF-*α* was determined after 2 h of LPS treatment.

### 2.8. NF-*κ*B DNA-Binding Activity

The DNA-binding activities of transcription factors in cells were determined by enzyme-linked DNA-protein interaction assay (ELDIA), according to a previously described method [21, 33]. Nuclear protein extracts were prepared using NE-PER Nuclear and Cytoplasmic Extraction Reagent as described previously [34]. DNA-p65 NF-*κ*B-binding activity was measured with a BD Mercury TransFactor kit (BD Bioscience, Clontech), which detects DNA binding by specific transcription factors, according to the manufacturer's instructions [35].

### 2.9. Western Blot Analysis

After treatment with indicated agents, cells were collected and lysed to determine expression of nNOS, iNOS, COX-2, gp91^phox^, I*κ*B*α* and HO-1. The lysates were centrifuged at 15 000 × g for 30 min at 4°C. The supernatant was then collected for SDS-polyacrylamide gel electrophoresis. Protein concentration was determined with the Bio-Rad protein assay kit following the manufacturer's guide. Cell membranes were obtained by ultracentrifugation of the supernatant at 26 000 × g for 1 h at 4°C. Equal amounts of protein (20 *μ*g per lane) were separated on a 10% polyacrylamide gel and transferred to polyvinylidene difluoride membranes (PerkinElmer Life and Analytical Sciences). Non-specific binding was blocked with TBST (50 mM Tris- HCl, pH 7.6, 150 mM NaCl, 0.1% Tween 20) containing 5% non-fat milk for 1 h at room temperature. The membranes were then each incubated overnight at 4°C with one of the following specific primary antibodies: mouse anti-iNOS (1 : 1000), mouse anti-gp91^phox^ (1 : 1000), mouse anti-*β*-actin (1 : 20 000), rabbit anti-nNOS (1 : 1000), mouse anti-I*κ*B*α* (1 : 500), rabbit anti-HO-1 (1 : 1000) and goat anti-COX-2 (1 : 5000). Membranes were washed six times for 5 min with TBST. The appropriate dilutions of secondary antibodies (diluted 1 : 1000) were incubated for 1 h. After six washes with TBST, the protein bands were detected with the enhanced chemiluminescence reagent (PerkinElmer Life and Analytical Sciences).

### 2.10. Measurement of Intracellular Reactive Oxygen Species

The level of intracellular reactive oxygen species (ROS) was quantified by fluorescence with H_2_DCF-DA. Following incubations with the indicated treatments, microglial BV-2 cells or neuronal SH-SY5Y cells were loaded with 10 *μ*M of H_2_DCF-DA for 30 min at 37°C. Cells were then washed three times with phosphate-buffer saline, pH 7.4, and the relative levels of fluorescence were analyzed by a spectrophotofluorimeter (FLUOstar OPTIMA, BMG LABTECH, Germany, 495 nm excitation and 520 nm emission). Intracellular ROS-containing cells were identified as those with increased FITC fluorescence of oxidized H_2_DCF.

### 2.11. Measurement of Mitochondrial Membrane Potential

Mitochondrial membrane potential was measured by the incorporation of a cationic fluorescent dye rhodamine 123. The reduction in fluorescent intensity of rhodamine 123 staining represented a fall in the mitochondrial membrane potential. After 24 h of incubation in normal medium with or without treatment, the cells were changed to serum-free medium containing 10 *μ*M rhodamine 123, there they were incubated for 15 min at 37°C. The cells were then collected, and the fluorescence intensity was analyzed within 15 min by a spectrophotofluorimeter (FLUOstar OPTIMA, BMG LABTECH, Germany, 490 nm excitation and 515 nm emission).

### 2.12. Data Analysis

Data were expressed as mean ± SEM. Analysis of variance (ANOVA) was used to assess the statistical significance of the differences followed by Tukey's test for all pair's comparisons. A value of *P* <  .05 was considered statistically significant. The data were analyzed with the Statistical Package for Social Sciences (SPSS, Chicago, IL).

## 3. Results

### 3.1. Effects of SHXT on LPS-Activated Microglial BV-2 Cell- and 6-OHDA-Induced Loss of SH-SY5Y Cell Viability

**Figure 1:**
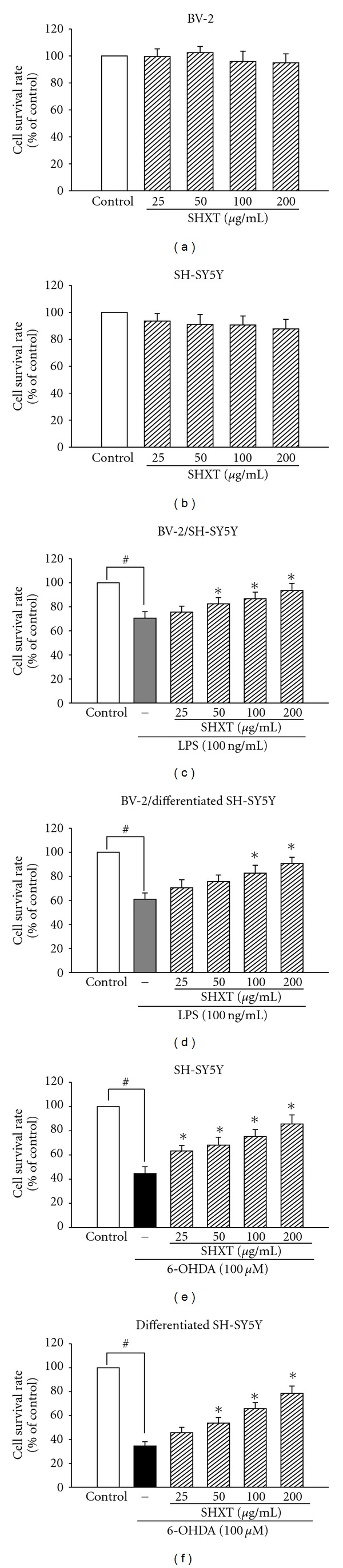
Effects of SHXT on microglial BV-2 cells (a), neuroblastoma SH-SY5Y cells (b), lipopolysaccharide (LPS, 100 ng/mL) activated BV-2 treated non-differentiated (c) and differentiated (d) SH-SY5Y cells, and 6-hydroxydopamine (6-OHDA, 100 *μ*M) treated non-differentiated (e) and differentiated (f) SH-SY5Y cells. Cells were treated with SHXT (25–200 *μ*g/mL) for 24 h. Cell viability was determined by MTT assay. Bars represent the mean ± SEM from six independent experiments. ^#^ *P* <  .05 versus control, *P <  .05 versus LPS or 6-OHDA only.

Cells were incubated with or without SHXT for 24 h. According to the results of MTT assay, SHXT (25, 50, 100 and 200 *μ*g/mL) did not effect the cell viability of BV-2 cells (Figure 1(a)) and SH-SY5Y cells (Figure 1(b)). In order to examine the effect of SHXT on LPS-activated microglia-mediated cell viability, co-culture of BV-2 cells with non-differentiated or differentiated SH-SY5Y cells was investigated. As shown in Figures 1(c) and 1(d), SHXT attenuated LPS (100 ng/mL)-activated BV-2 cells-induced cell death in non-differentiated and differentiated SH-SY5Y cells. Moreover, exposure of non-differentiated (Figure 1(e)) and differentiated (Figure 1(f)) SH-SY5Y cells to 6-OHDA induced significant cell death. SHXT increased cell viability in 6-OHDA treated non-differentiated (Figure 1(e)) and differentiated (Figure 1(f)) SH-SY5Y cells. SHXT protected against activated microglia- and 6-OHDA-induced differentiated neuronal cell death at the concentrations from 100 to 200 *μ*g/mL and 50 to 200 *μ*g/mL, respectively. In addition, we examined dopamine receptor expression of differentiated SH-SY5Y cells using western blotting, and marked increase of dopamine receptor expression was observed (online Supplementary FigureS1).

### 3.2. LPS-Induced Inflammation and Associated Oxidative Stress-Related Factors in BV-2 Cells were Reduced by SHXT

**Figure 2:**
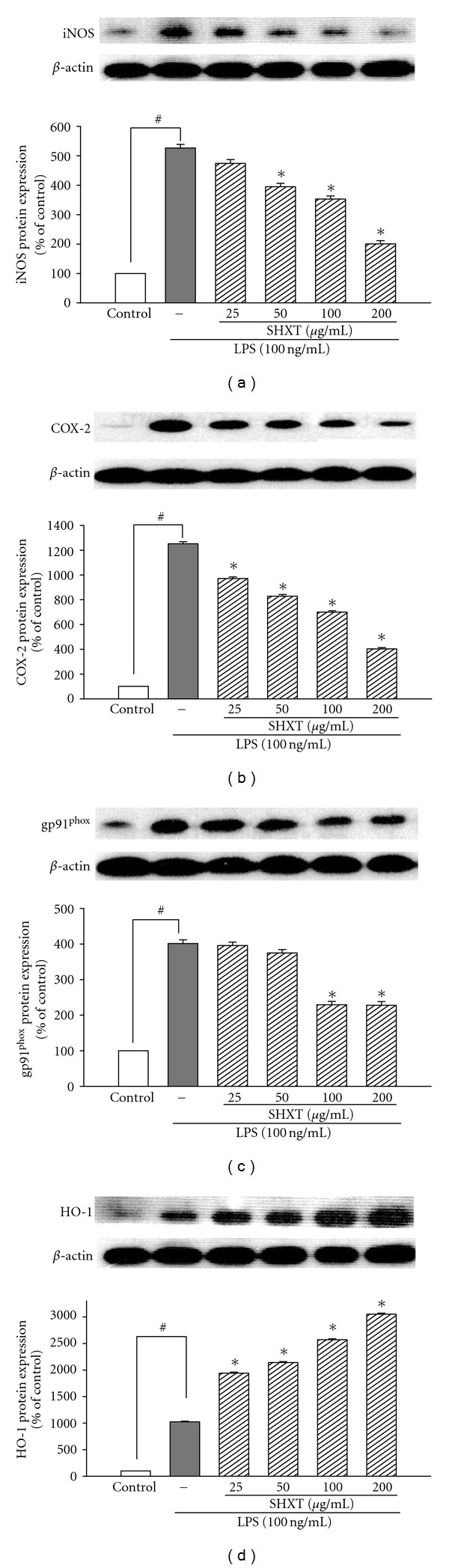
Effects of SHXT on expression of iNOS, COX-2, gp91phox and HO-1 in BV-2 cells treated with lipopolysaccharide (LPS, 100 ng/mL) for 24 h. Cultures were pretreated with SHXT for 30 min before the addition of LPS treatment. Bars represent the mean ± SEM from six independent experiments. Densitometry analyses are presented as the relative ratio of protein/*β*-actin protein, and are represented as percentages of the control group. ^#^ *P* <  .05 versus control, *P <  .05 versus LPS only.

**Figure 3:**
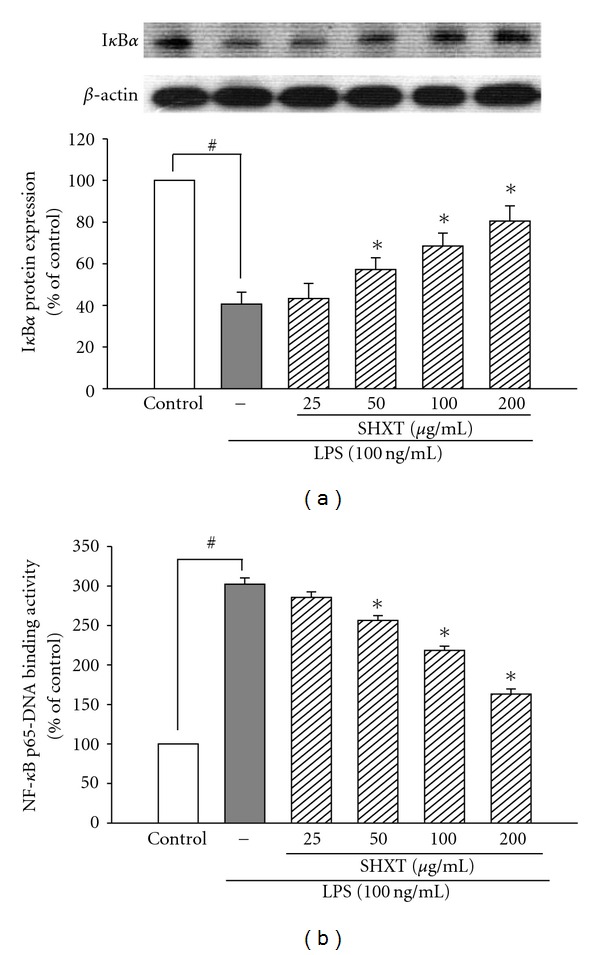
Effects of SHXT on the I*κ*B*α* expression (a) and NF-*κ*B activation (b) in lipopolysaccharide (LPS, 100 ng/mL)-treated BV-2 cells. Cultures were pretreated with SHXT for 30 min before the addition of LPS. Then the cells were collected at 1 h for NF-*κ*B activity assay and at 24 h for I*κ*B*α* protein analyses. Densitometry analyses are presented as the relative ratio of protein/*β*-actin protein, and they are represented as percentages of the control group. Changes of NF-*κ*Bp65 translocation levels are represented as percentages of the control group. Bars represent the mean ± SEM from six independent experiments. ^#^ *P* <  .05 versus control, *P <  .05 versus LPS only.

To investigate whether SHXT inhibited LPS-activated microglia-induced inflammatory and oxidative stress factors, the effects of SHXT on the associated noxious factors of LPS-treated BV-2 cells were examined. As shown in Figure 2, LPS (100 ng/mL) increased expression of iNOS, COX-2 and gp91^phox^ in BV-2 cells, which were inhibited by SHXT treatment. Pairwise comparison at each concentration by Tukey's test indicated that SHXT concentration-dependently inhibited iNOS at a concentration of 50 and 200 *μ*g/mL and inhibited COX-2 at a concentration of 25, 50, 100 and 200 *μ*g/mL. Additionally, SHXT upregulated HO-1 expression in LPS treated BV-2 cells in a concentration-dependent manner from 25 to 200 *μ*g/mL (Figure 2(d)). SHXT also reduced LPS-induced NO, PGE_2_ and iROS production (Table 1). Furthermore, SHXT (50–200 *μ*g/mL) concentration-dependently attenuated I*κ*B*α* degradation and NF-*κ*Bp65 translocation in LPS-treated BV-2 cells (Figure 3).

### 3.3. SHXT Inhibited Overproduction of TNF-*α* and IL-1*β* in LPS-Treated BV-2 Cells and Overexpression of nNOS, COX-2 and gp91^phox^ in SH-SY5Y Cells Co-Cultured with BV-2 Cells

**Figure 4:**
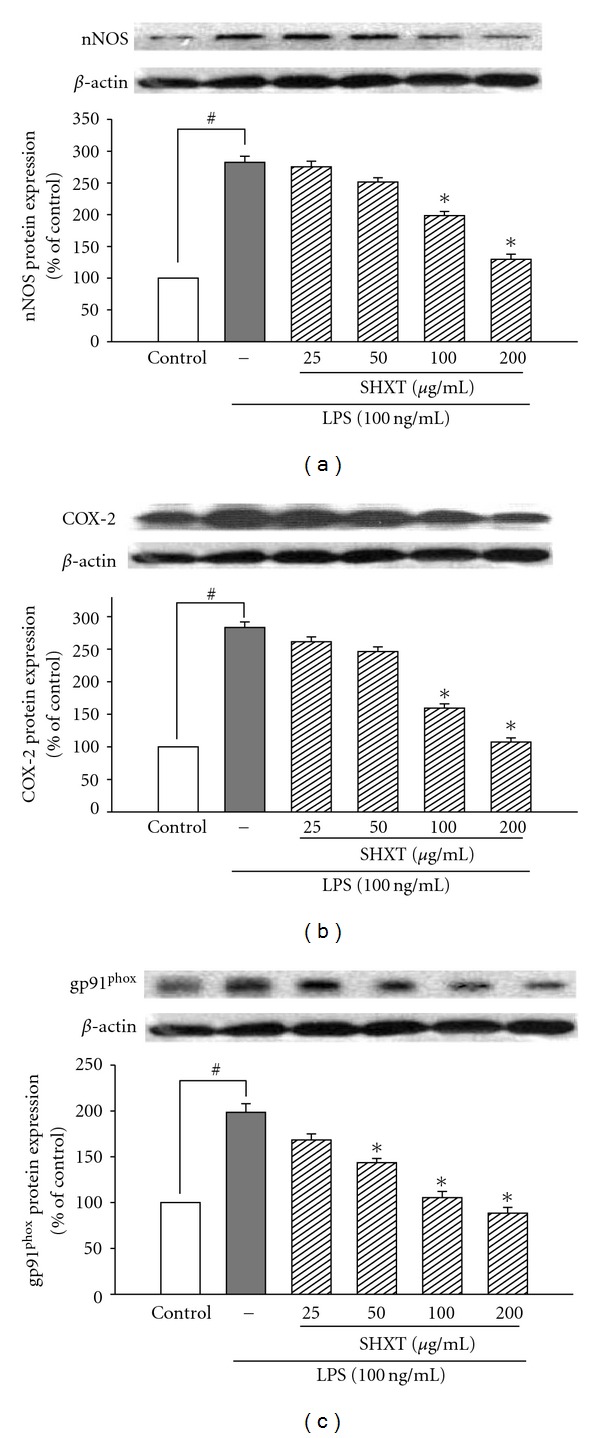
Inhibitory effects of SHXT on the expressions of nNOS (a), COX-2 (b) and gp91^phox^ (c) in SH-SY5Y cells under co-culture with lipopolysaccharide (LPS, 100 ng/mL)-treated BV-2 cells. Cultures were pretreated with SHXT for 30 min followed by LPS treatment for 24 h. Densitometry analyses are presented as the relative ratio of protein/*β*-actin protein, and are represented as percentages of the control group. Bars represent the mean ± SEM from six independent experiments. ^#^ *P* <  .05 versus control, *P <  .05 versus LPS only.

As shown in Table 1, LPS (100 ng/mL) caused a marked increase of TNF-*α* at 2 h and IL-1*β* at 24 h in BV-2 cells. Results indicated SHXT concentration-dependently attenuated LPS-induced overproduction of TNF-*α* and IL-1*β* from 25 to 200 *μ*g/mL and 50 to 200 *μ*g/mL, respectively. Under co-culture conditions, LPS exposure induced upregulation of nNOS, COX-2 and gp91^phox^ of SH-SY5Y cells. However, SHXT (50–200 *μ*g/mL) concentration-dependently attenuated LPS-activated BV-2 cell-induced overexpression of nNOS (Figure 4(a)), COX-2 (Figure 4(b)) and gp91^phox^ (Figure 4(c)) on SH-SY5Y cells.

### 3.4. 6-OHDA-Induced Inflammatory Factors and Mitochondrial Membrane Potential Changes in SH-SY5Y Cells Were Attenuated by SHXT

**Figure 5:**
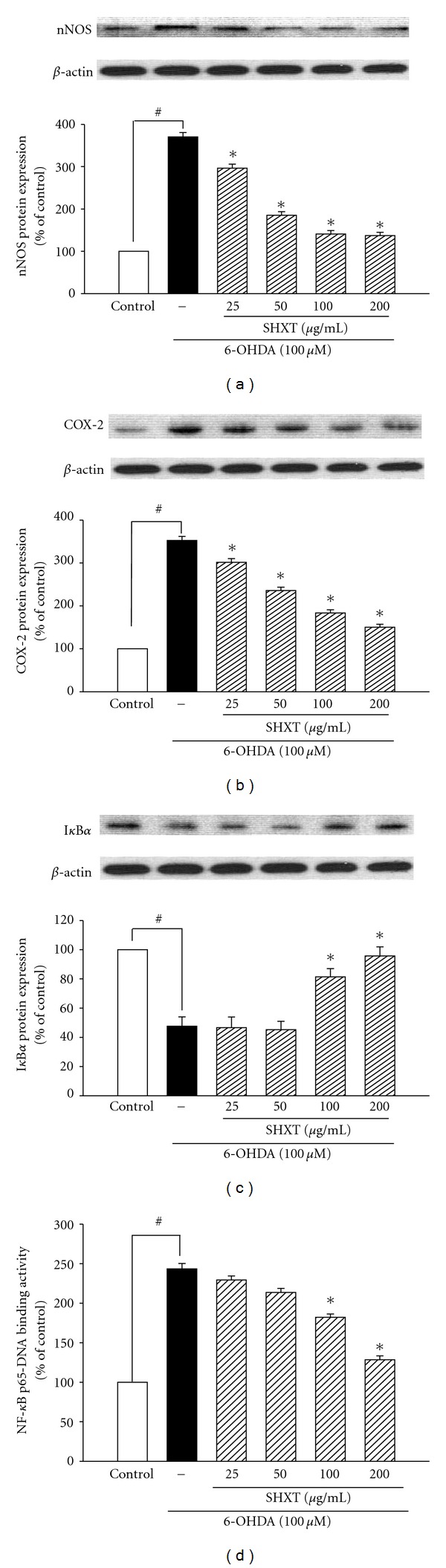
Inhibitory effects of SHXT on the expressions of nNOS (a), COX-2 (b), I*κ*B*α*(c) and NF-*κ*B activation (d) on 6-hydroxydopamine (6-OHDA, 100 *μ*M) treated SH-SY5Y cells. Cultures were pretreated with SHXT for 30 min before the addition of 6-OHDA. Then the cells were collected at 1.5 h for NF-*κ*B activity assay and at 24 h for nNOS, COX-2 and I*κ*B*α* protein analyses. Densitometry analyses are presented as the relative ratio of protein/*β*-actin protein, and are represented as percentages of the control group. Changes of NF-*κ*B p65 translocation levels are represented as percentages of the control group. Bars represent the mean ± SEM from six independent experiments. ^#^ *P* <  .05 versus control, *P <  .05 versus 6-OHDA only.

**Figure 6:**
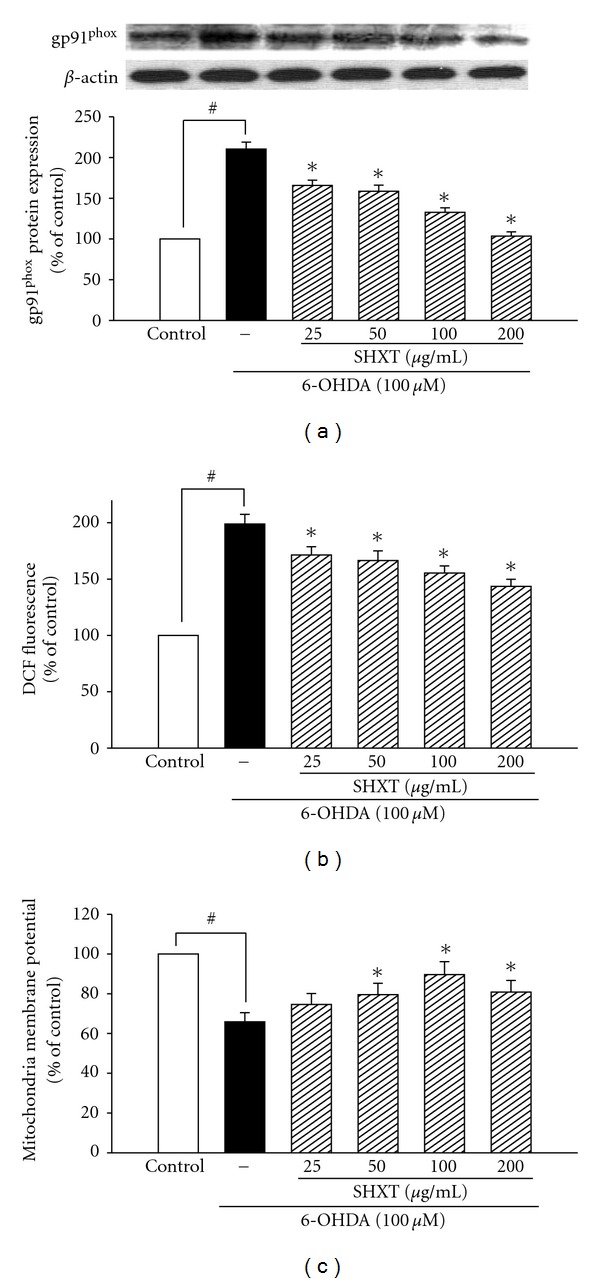
Effects of SHXT on 6-hydroxydopamine (6-OHDA, 100 *μ*M)-induced changes of gp91^phox^ expression (a), iROS formation (b) and mitochondria membrane potential (c) in SH-SY5Y cells. The iROS was measured by using H2DCF-DA staining and mitochondrial membrane potential was measured by rhodamine 123. Densitometry analyses are presented as the relative ratio of protein/*β*-actin protein, and are represented as percentages of the control group. Bars represent the mean ± SEM from six independent experiments. ^#^ *P* <  .05 versus control group, *P <  .05 versus 6-OHDA only.

SH-SY5Y cells treated with 6-OHDA (100 *μ*M) for 24 h resulted in significant increases in expression of nNOS (Figure 5(a)) and COX-2 (Figure 5(b)), which were inhibited by SHXT (25–200 *μ*g/mL). SHXT also inhibited 6-OHDA-induced I*κ*B*α* degradation (Figure 5(c)) and NF-*κ*Bp65 translocation (Figure 5(d)) at a concentration of 100 *μ*g/mL and 200 *μ*g/mL. SHXT (25–200 *μ*g/mL) attenuated 6-OHDA-induced increases of gp91^phox^ (Figure 6(a)) and iROS (Figure 6(b)). Furthermore, SHXT (50–200 *μ*g/mL) significantly attenuated 6-OHDA-induced decrease in mitochondria membrane potential (Figure 6(c)).

## 4. Discussion

**Figure 7:**
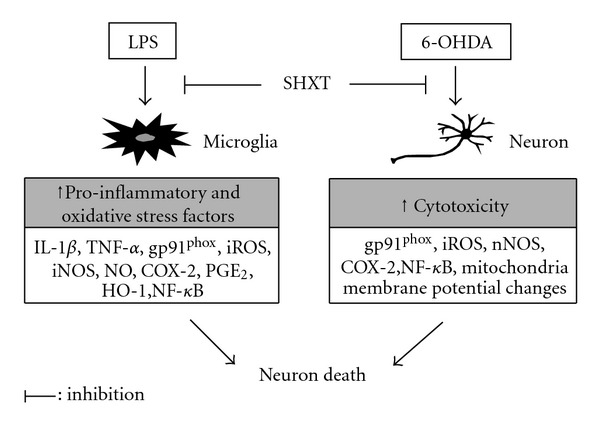
Hypothetical protective mechanisms of SHXT on microglia-mediated and 6-OHDA-induced toxicity in neuronal cells.

The present study demonstrated that SHXT could inhibit LPS-activated microglia- and neurotoxin 6-OHDA-induced neurotoxicity. The major findings showed that SHXT significantly reduced LPS-activated microglia- and 6-OHDA-induced neuronal cell death. In LPS-activated microglia-like BV-2 cells, SHXT attenuated inflammatory cytokine TNF-*α* and IL-1*β*, decreased the production of NO, PGE_2_ and iROS, downregulated overexpression of iNOS, COX-2 and gp91^phox^, decreased I*κ*B*α* degradation and NF-*κ*Bp65 translocation, and upregulated HO-1 expression. Moreover, SHXT downregulated overexpression of nNOS, COX-2, gp91^phox^ on neuronal SH-SY5Y cells co-cultured with activated microglia BV-2 cells. In 6-OHDA-induced neurotoxicity, the protective effects of SHXT are mediated by downregulating nNOS, COX-2 and gp91^phox^, decreased iROS level, I*κ*B*α* degradation and NF-*κ*Bp65 translocation and increased mitochondria membrane potential of SH-SY5Y cells (Figure 7).

Microglial-mediated neurotoxicity has been implicated in numerous neurodegenerative diseases [29]. Activated microglia increase oxidative stress [36] and inflammatory mediators [37]. Pro-inflammatory cytokines TNF-*α* and IL-1*β* are thought to play a major role in inducing neuronal death [38]. In this study, SHXT significantly attenuated LPS-induced increase of pro-inflammatory cytokines (TNF-*α* and IL-1*β*) on BV-2 cells. Moreover, pro-inflammatory cytokines can induce iNOS expression and subsequently induce large amounts of NO production in activated microglia [7]. NO, together with iNOS and nNOS, is known to be involved in the pathogenesis of PD [39, 40]. Excessive accumulation of NO has long been known to be toxic to neurons [41]. Oxygen free radicals such as superoxide can react with NO to form much more deadly intermediates such as peroxynitrite [42]. The current results indicated that SHXT not only reduced LPS-activated microglia-induced increase of iNOS and NO, but also decreased the overexpression of nNOS in microglia-stimulated neuronal SH-SY5Y cells. SHXT also attenuated the overexpression of nNOS induced by 6-OHDA in SH-SY5Y cells. COX-2 is believed to be a critical enzyme in the inflammatory response and has been implicated in neuronal survival and death [43]. Cytokines (particularly TNF-*α*) activate COX-2 enzymes, and inhibition of COX-2 has been shown to provide neuroprotection [44]. Our results showed that SHXT not only decreased TNF-*α* production but also attenuated COX-2 expression and PGE_2_ production on LPS-treated microglia BV-2 cells. SHXT also inhibited overexpression of COX-2 on activated microglia-treated or 6-OHDA-treated neuronal SH-SY5Y cells. It is well known that HO-1 can inhibit iNOS expression and NO production in activated macrophages via inactivation of NF-*κ*B [45, 46]. Our results showed that SHXT inhibited LPS-induced iNOS expression, NO production, I*κ*B*α* degradation and NF-*κ*B p65 translocation. SHXT also enhanced HO-1 expression.

Phagocyte NADPH oxidase (PHOX) is a major source of superoxide and the catalytic center of this oxidase is the membrane-integrated protein gp91^phox^ [47, 48]. NADPH oxidase is deeply involved with microglia-induced neurotoxicity as a result of either generating extracellular ROS or by increasing microglia intracellular ROS production, which activates the generation of pro-inflammatory mediators and subsequently induces damages to neurons [1]. Microglia are critical to NADPH oxidase-mediated neurotoxicity in LPS-induced inflammation-mediated neurodegeneration [49]. Our study showed that SHXT decreased gp91^phox^ overexpression and iROS levels on LPS-activated BV-2 cells. SHXT also downregulated gp91^phox^ overexpression on SH-SY5Y cells co-cultured with LPS-activated microglia BV-2 cells. Additionally, NADPH oxidase-generated ROS are involved in the signaling event leading to COX-2 expression and PGE_2_ production in microglia treated with LPS [50]. The present results indicated SHXT inhibited gp91^phox^, iROS, COX-2 and PGE_2_. Therefore, the protective effects of SHXT on LPS-activated microglia-induced neuronal cell damage might be due to its anti-inflammatory and anti-oxidative effects.

It has been reported that the auto-oxidation of dopamine to 6-OHDA generates ROS and reactive quinones [31, 32], which subsequently induces lipid peroxidation, damages mitochondrial membrane and results eventually in the collapse of mitochondrial membrane potential, leading to cell death [51]. The present results indicated that SHXT decreased gp91^phox^ overexpression and iROS levels in SH-SY5Y cells exposed to 6-OHDA. SHXT also attenuated the decrease of mitochondrial membrane potential and cell death induced by 6-OHDA. Furthermore, SHXT also inhibited 6-OHDA-induced increases of nNOS, COX-2, I*κ*B*α* degradation and NF-*κ*Bp65 translocation. Therefore, the prevention of SHXT on 6-OHDA-induced neuronal cell damage might be partly mediated via decreasing production of iROS, increasing mitochondrial membrane potential and attenuating overexpression of inflammatory protein.

In conclusion, this study suggests that SHXT can effectively attenuate LPS-activated microglia- and neurotoxin 6-OHDA-induced neurotoxicity. Considering the importance of inflammation and ROS in neurodegeneration, SHXT might have a protective potential in the microglia-mediated or oxidative stress-related neurodegenerative disorders.

## Supplementary Data

Supplementary data are available at *eCAM* online.

## Funding

National Science Council, Taiwan (NSC-96-2320-B-037-039-MY3).

## Supplementary Material

Dopamine receptor expression of differentiated SH-SY5Y cells using western blotting.

## Figures and Tables

**Table 1:** Effects of SHXT (25, 50, 100 and 200 *μ*g/mL) on LPS-induced nitrite, PGE_2_, iROS, TNF-*α* and IL-1*β* production in BV-2 cells.

	Nitrite (*μ*M)	PGE_2_ (pg/mL)	iROS (%)	TNF-*α* (pg/mL)	IL-1*β* (pg/mL)
Control	0.25 ± 0.03	14.45 ± 1.53	100	70.41 ±8. 51	10.51 ± 3.57
LPS only	3.76 ± 0.20^#^	41.83 ± 2.53^#^	201.52 ± 9.52^#^	704.61 ± 30.53^#^	68.51 ± 5.65^#^
LPS + SHXT 25 *μ*g/mL	2.96 ± 0.41	27.39 ± 2.14*	192.51 ± 8.52	650.25 ± 20.56	58.52 ± 7.24
LPS + SHXT 50 *μ*g/mL	1.89 ± 0.34*	22.52 ± 1.95*	182.57 ± 8.50	552.53 ± 10.51*	50.14 ± 4.65*
LPS + SHXT 100 *μ*g/mL	1.63 ± 0.21*	21.62 ± 1.52*	130.73 ± 6.25*	430.56 ± 12.86*	42.51 ± 4.64*
LPS + SHXT 200 *μ*g/mL	1.50 ± 0.14*	14.68 ± 1.35*	109.64 ± 6.63*	263.50 ± 15.58*	26.52 ± 5.63*

Values represent the mean ± SEM from six independent experiments. ^#^ *P* <  .05 versus control, *P <  .05 versus LPS only.
