# Supplementary figures and images for: San-Huang-Xie-Xin-Tang Protects against Activated Microglia- and 6-OHDA-Induced Toxicity in Neuronal SH-SY5Y Cells
Source: Evid Based Complement Alternat Med. 2011 Jan 4;2011:429384. doi: 10.1093/ecam/nep025 (PMC3135633; doi:10.1093/ecam/nep025)

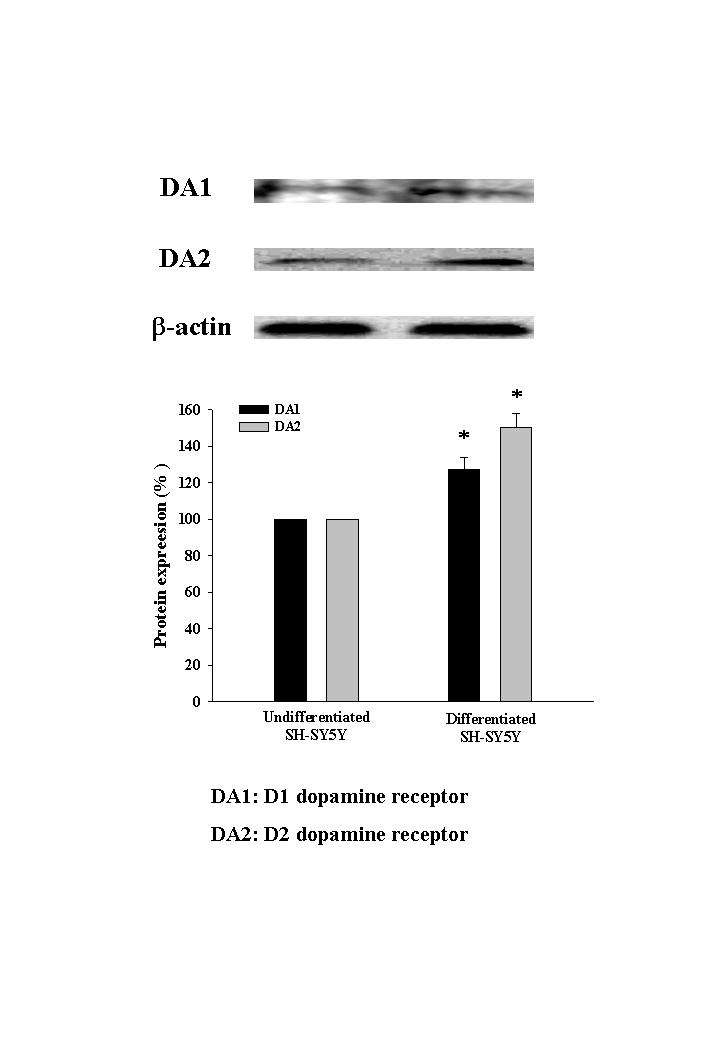

Supplement: Supplementary file 1 — Dopamine receptor expression of differentiated SH-SY5Y cells using western blotting. [file 429384.f1.jpg]
